# New distributional records of Southern Ocean Isopoda based on vouchers from the Italian National Antarctic Museum (MNA, Section of Genoa)

**DOI:** 10.3897/BDJ.12.e127689

**Published:** 2024-07-17

**Authors:** Nicholas Noli, Matteo Cecchetto, Alice Guzzi, Marco Grillo, Valentina Cometti, Stefano Schiaparelli

**Affiliations:** 1 Department of Physical Sciences, Earth and Environment (DSFTA) - University of Siena, Siena, Italy Department of Physical Sciences, Earth and Environment (DSFTA) - University of Siena Siena Italy; 2 Italian National Antarctic Museum (MNA, section of Genoa), Genoa, Italy Italian National Antarctic Museum (MNA, section of Genoa) Genoa Italy; 3 Department of Earth, Environmental and Life Sciences (DISTAV) - University of Genoa, Genoa, Italy Department of Earth, Environmental and Life Sciences (DISTAV) - University of Genoa Genoa Italy

**Keywords:** biogeography, museum collection, Ross Sea, Southern Ocean, Terra Nova Bay

## Abstract

**Background:**

The order Isopoda Latreille, 1816 consists of species occurring in terrestrial, marine and freshwater environments. In the Southern Ocean (SO), this group is amongst the most speciose and occur at all depths. Isopoda biogeography, despite being studied since the first Antarctic expeditions, is still poorly known from a geographical point of view and shows large occurrence gaps for some groups in specific sectors of the SO. In this paper, we update the isopod checklists of the Ross Sea (RS) and of some peri-Antarctic areas, such as the South Orkney Islands (SOI) and the South Sandwich Islands (SSI), based on the study of museum vouchers curated by the Italian National Antarctic Museum (MNA, Section of Genoa).

**New information:**

A total of 279 MNA samples from 15 different expeditions were studied. From this material, consisting of 419 specimens, 41 accepted species distributed in 24 families and 51 genera were identified. Comparing this newly-obtained information with the GBIF (Global Biodiversity Information Facility) and OBIS (Ocean Biodiversity Information System) portal, 15 species are here reported for the first time in the Ross Sea, with five new records in the Ross Sea Marine Protected Area. All records are new for the Terra Nova Bay area, for which a checklist of this group has never been produced before.

## Introduction

Isopoda Latreille, 1816 contains 10,740 accepted species (World Register of Marine Species, accessed on 24/06/2024) occurring in terrestrial, marine and freshwater environments (https://www.marinespecies.org/isopoda/aphia.php?p=stats); marine species, which account for nearly for two-thirds of all described isopods (6,276 accepted species according World Register of Marine Species accessed 24/06/2024), are fairly well known (Ahyong et al. 2024). However, new-to-science species are continuously described from all depths, suggesting that their inventory is still far from complete. In the Southern Ocean, isopods represent a highly speciose group (Kussakin 1967, Broyer et al. 2003). In terms of species richness and abundance, they play a key role in structuring SO benthic communities (Brandt 1999, Clarke and Johnston 2003), occurring at all depths, from the shelf to the deep sea (Brandt et al. 2004) and representing 7.1% (441 species) of the totality of the isopod species described so far (Kaiser 2014).

From a biogeographic point of view, the scientific campaigns carried out in the SO confirmed the importance of isopods in the area and adding new species descriptions and new distributional records.

In this paper, we deal with distributional records of species from three areas: i) the Ross Sea (RS); ii) the South Orkney Islands (SOI) and iii) the South Sandwich Islands (SSI).

In the framework of the XIX^th^ Italian Antarctic Expedition held in RS in 2004 on board the RV *Italica*, isopods retrieved through fine mesh dredge sampling (Rauschert dredge with a 500 μm mesh size) represented the second most abundant group amongst benthic Arthropoda. In fact, out of the 47,871 Peracarida specimens collected, 9,494 were Isopoda, representing the 23% of the sampled peracarids and the third most abundant group of the totality of macrozoobenthos sampled (with a relative frequency percentage of 14.61%) (Rehm et al. 2006). The PNRA XIX^th^ Italian Antarctic Expedition, part of the Victoria-Land Latitudinal Gradient Project (Berkman et al. 2005), was the first time in which a fine mesh sampling gear (Rauschert dredge) was used. This expedition sampled 117 isopod species, distributed in 20 families and in 49 genera (Choudhury and Brandt 2007, Choudhury and Brandt 2009). The RS area is a very important area of the SO, being the largest Marine Protected Area in the world and was created in 2017 to preserve the high species diversity and unique pristine condition (CCAMLR 2016 Delegations of New Zealand and the USA 2016). It is, therefore, important to focus on isopods in order to reach an understanding of isopod species distributions and presence in RS.

The South Orkney Islands (SOI) marine macrozoobenthos has been studied during many expeditions since the late 1800s (Barnes et al. 2009) and compared, in recent works, with that of the Amundsen Sea (Barnes 2008). The British Antarctic Survey (BAS) JR 144 and JR 179 cruises, held in the framework of the projects BIOPEARL 1 and BIOPEARL 2, with the RRS *James Clark Ross* (Kaiser et al. 2009), used an epibenthic sledge (EBS) to make quantitative comparisons between the two areas. Results indicated that, out of the total number of isopod species found, 66.7% of those recovered from the Scotia Sea shelf and 93.8% of those recovered from the Scotia Sea slope and deep sea were still undescribed (Kaiser et al. 2009). EBS was used in SOI also in recent times, showing the isopods as the second most abundant order in the area (Di Franco et al. 2020).

Furthermore, is important to mention some examples of new species of isopods retrieved in the SOI area, for example, *Pseudidotheaarmata* Noli, Di Franco, Schiaparelli & Brandt, 2022 (Noli et al. 2022) (present in this dataset).

Epibenthic sledge (EBS) sampling was also performed in the marine area of the South Sandwich Islands (SSI), an archipelago of volcanic islands. A pattern of decreasing richness and abundance with increasing depth has been recently uncovered from the shelf of the Sandwich Islands to the deep zone, in the framework of BAS JR 144 BIOPEARL 1 (Kaiser et al. 2008). A study of isopod abundances using EBS sampling in the SSI area was conducted in November 1994 in the framework of the Joint Magellan "Victor Hansen" Campaign, showing a high level of species endemism (31%) (Brandt et al. 1999).

In this paper, we update the isopod checklists of RS and of some peri-Antarctic areas, such as SOI and the SSI, based on the study of museum vouchers deposited in the Italian National Antarctic Museum (MNA, Section of Genoa). Samples were collected in the framework of 15 Antarctic Expeditions and compared with Global Biodiversity Information System (GBIF) occurrences of isopods. A total of 339 isopod species in the SO, 71 in RS area, 115 in the SOI and 59 in the SSI area (GBIF.Org 2023b) were identified.

The aim of this work was to increase species distribution knowledge in the studied areas thanks to the availability of newly-collected materials curated by the MNA. This dataset is an MNA contribution to the Antarctic Biodiversity Portal, the thematic Antarctic node for both the Ocean Biogeographic Information System (AntOBIS) and the Global Biodiversity Information Facility (ANTABIF). Previous MNA contributions focused on Mollusca, Tanaidacea, Fungi, Ophiuroidea, Porifera, Bryozoa, Rotifera, Asteroidea and Copepoda (Ghiglione et al. 2013, Piazza et al. 2014, Selbmann et al. 2015, Cecchetto et al. 2017, Ghiglione et al. 2018, Cecchetto et al. 2019, Garlasché et al. 2019, Bonello et al. 2020, Guzzi et al. 2022) and the current special issue includes additional articles that focus on other organisms, such as copepods (Grillo et al. 2024), holothuroids (Guzzi et al., in prep.), amphipods (Cecchetto et al., in prep.), ARMS fouling (Cometti et al., in prep.) and fishes. This dataset also represents another Italian contribution to the CCAMLR CONSERVATION MEASURE 91-05 (2016) for the Souther Ocean, with a focus on the Ross Sea region Marine Protected Area, specifically addressing Annex 91-05/C (“long-term monitoring of benthic ecosystem functions”).

## General description

### Purpose

The purpose of this data paper is to provide information and data about the Italian National Antarctic Museum's (MNA, Section of Genoa) Isopoda collection. MNA samples were collected in the framework of 15 different Antarctic research expeditions: PNRA Expedition X (1994/95), XI (1995/96), XIII (1997/98), XIV (1998/99), XVII (2001/02), XIX (2003/04), XXV (2009/10), XXVII (2011/12), XXVIII (2012/13), XXIX (2013/14), XXXII (2016/17), XXXIV (2018/19) and NSF (National Science Foundation) "Icefish04" and BAS (British Antarctic Survey) Expeditions JR15005 "SO-AntEco" and JR18003 "ICEBERGS2".

## Sampling methods

### Study extent

This study was focused on the voucher specimens of the Isopoda collection curated by the Italian National Antarctic Museum (MNA, Section of Genoa).

### Sampling description

Sampling methods in this dataset vary from cruise to cruise: triangular dredge (IX, XVII PNRA exp.), large dredge (XXV, XXVII, XXVIII, XXIX PNRA exp.), Rauschert dredge (XIX PNRA exp., BAS JR15005 "SO-AntEco" 2016 exp.), Agassiz trawl (AGT) (XIII PNRA exp., XIX PNRA exp., BAS JR18003 "ICEBERGS2" 2018 exp.), Multiple Net Tucker Trawl (TT) (XXIX PNRA exp.), gill net (GN) (XXVII, XXVIII PNRA exp.), Small Hamburg Plankton Net (SHPN) (XXVIII PNRA exp.), trammel net (TN) (XXIX PNRA exp.), 65l Van Veen grab (IX, XI, XIV, XVII XIX PNRA exp.), box corer and multi corer (XXXII PNRA exp.), long line (LL) (XXVIII PNRA exp.) and manual collection through SCUBA diving (XVII, XXV, XXXIV PNRA exp.).

### Quality control

Damaged and data-deficient samples were excluded from this dataset. Coordinates were verified, plotted on a map and cross-checked with the original data logs. All scientific names were matched to the AphiaID at the World Register of Marine Species (Ahyong et al. 2024). The event date and time were formatted in ISO 8601.

### Step description

All the available samples from the MNA collection were classified to the lowest possible taxonomic level, on a morphological basis, by observing their diagnostic characters with a Leica MZ8 optical stereomicroscope. The R package "rgbif" (version 3.7.5) was used to retrieve existing GBIF distributional records (Chamberlain et al. 2023) from different areas: the whole SO (GBIF.Org 2023a), the South Orkney Islands (GBIF.Org 2023b) and the South Georgia and the South Sandwich Islands areas (GBIF.Org 2023c). The OBIS database (Intergovernmental Oceanographic Commission of UNESCO 2023) and bibliographic research were also used in order to confirm new records in the given study areas. QGIS (QGIS.org, QGIS Geographic Information System, QGIS Association) and the layers repository Quantarctica (Matsuoka et al. 2021) were used to produce occurrence maps and GIS-related biogeographical information. In order to give a spatial delimitation and to confirm new records for the given study areas, CCAMLR Statistical Areas, subareas and division (CCAMLR 1980) were used.

## Geographic coverage

### Description

**Figure 1.**
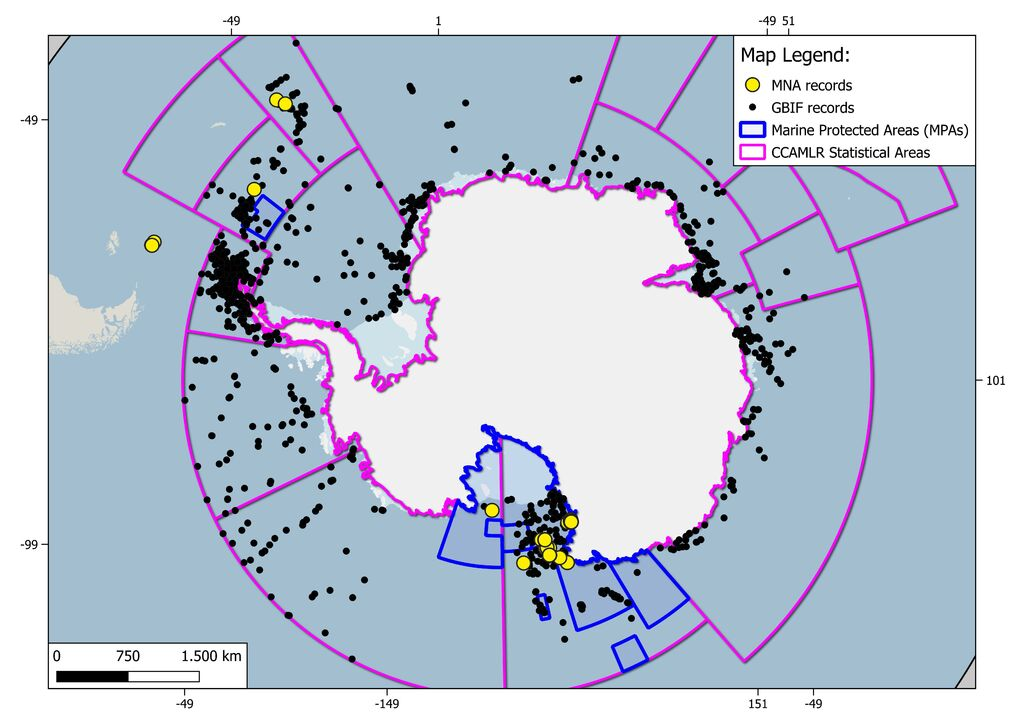
Overview of the Southern Ocean area isopod records of Italian National Antarctic Museum (MNA, Section of Genoa); MNA records are marked with yellow dots and Global Biodiversity Information System (GBIF) database records (accessed on 28 February 2023) are marked with black dots. CCAMLR (Convention on the Conservation of Antarctic Marine Living Resources) Statistical Areas, subareas and divisions are shown as purple lines, while Marine Protected Areas are represented as blue lines.

**Figure 2.**
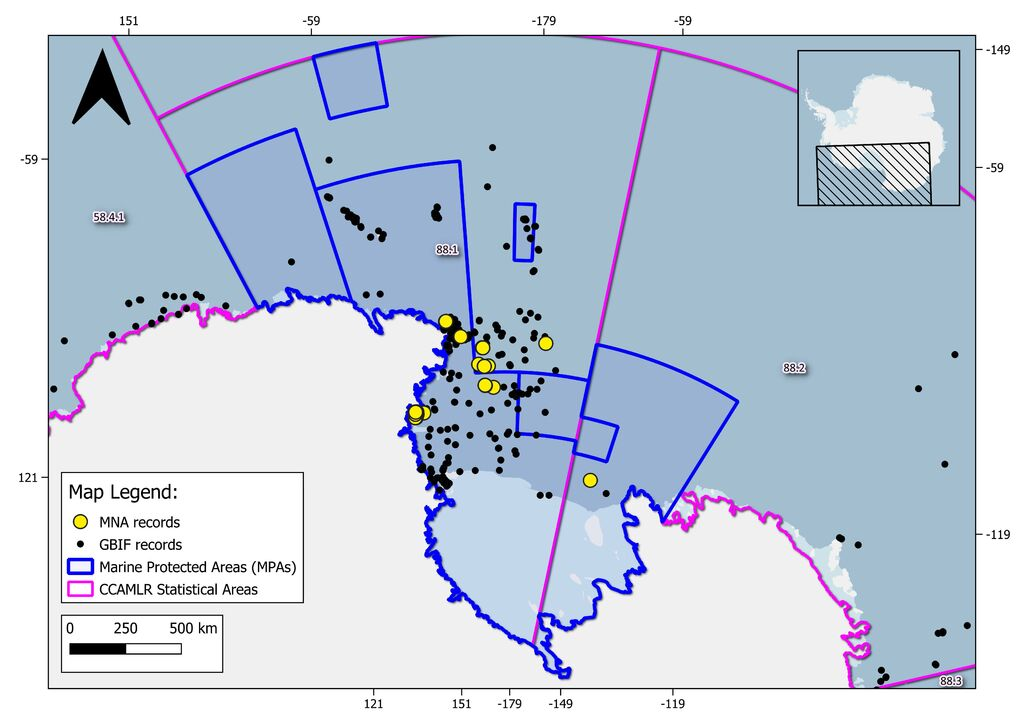
Overview of the Marine Protected Areas (MPAs) present in the Ross Sea; Italian National Antarctic Museum (MNA, Section of Genoa) isopod records are marked with yellow dots and Global Biodiversity Information System (GBIF) database records (accessed on 28 February 2023) are marked with black dots. CCAMLR (Convention on the Conservation of Antarctic Marine Living Resources). Statistical Areas, subareas and divisions are shown as purple lines, while MPAs are represented as blue lines.

**Figure 3.**
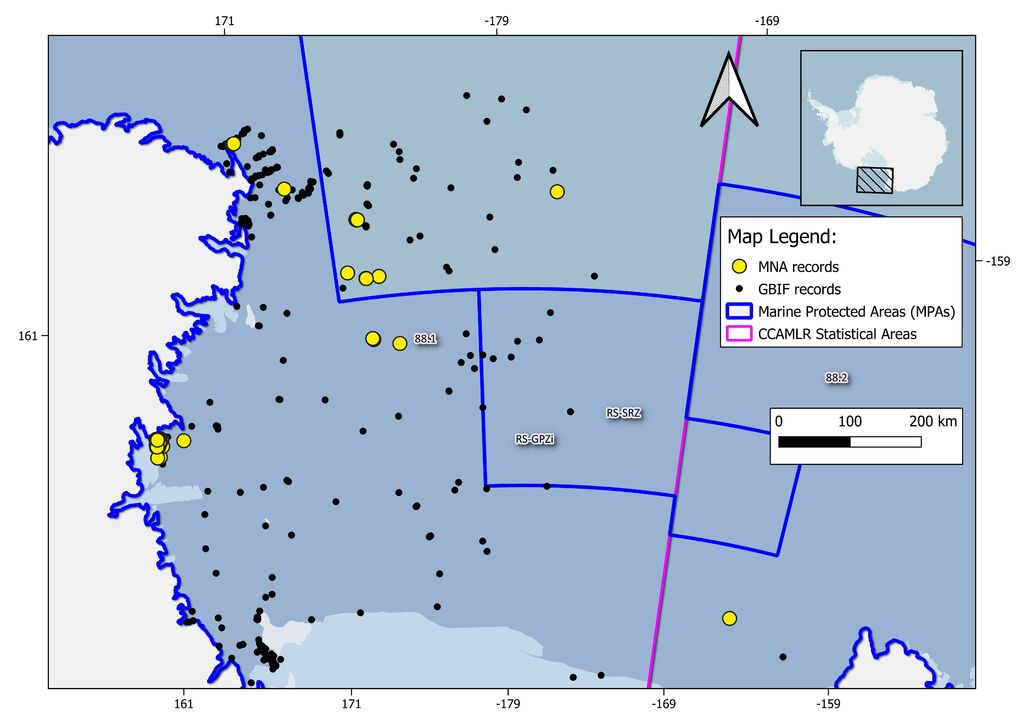
Highlight of the Ross Sea area isopod records; Italian National Antarctic Museum (MNA, Section of Genoa) records are marked with yellow dots and Global Biodiversity Information System (GBIF) database records (accessed on 28 February 2023) are marked with black dots. CCAMLR (Convention on the Conservation of Antarctic Marine Living Resources) Statistical Areas, subareas and divisions are shown as purple lines, while Marine Protected Areas are represented as blue lines.

**Figure 4.**
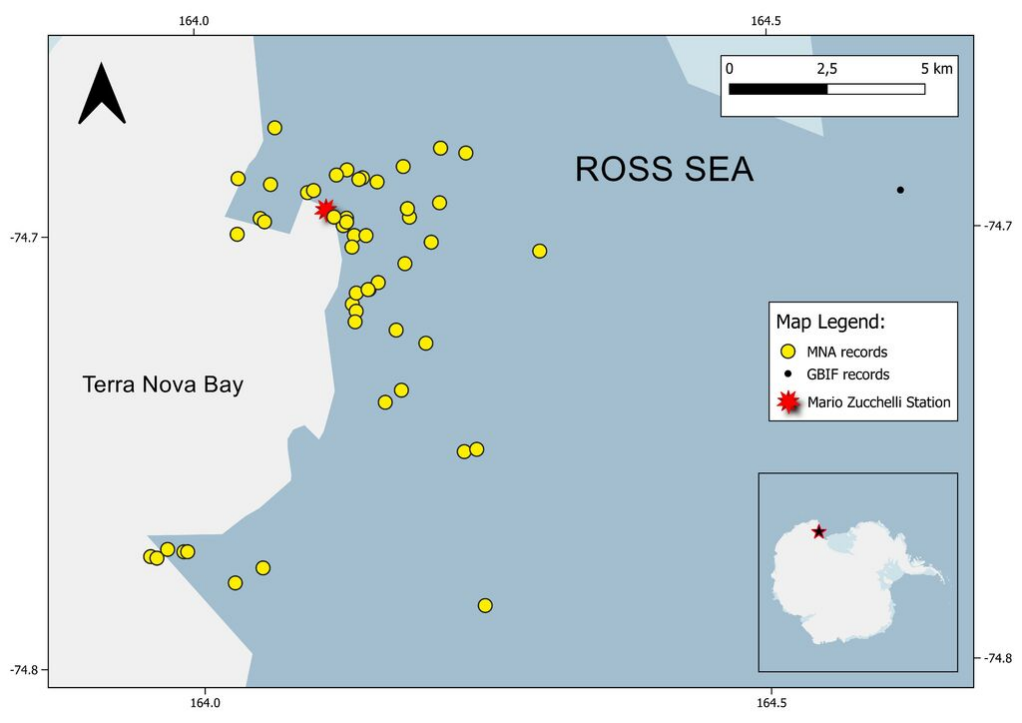
Focus of Terra Nova Bay Ross Sea area records; Italian National Antarctic Museum (MNA, Section of Genoa) records are marked with yellow dots and Global Biodiversity Information System (GBIF) database records (accessed in 28 February 2023) are marked with black dots. Mario Zucchelli Italian Research Station is marked here with a red star.

**Figure 5.**
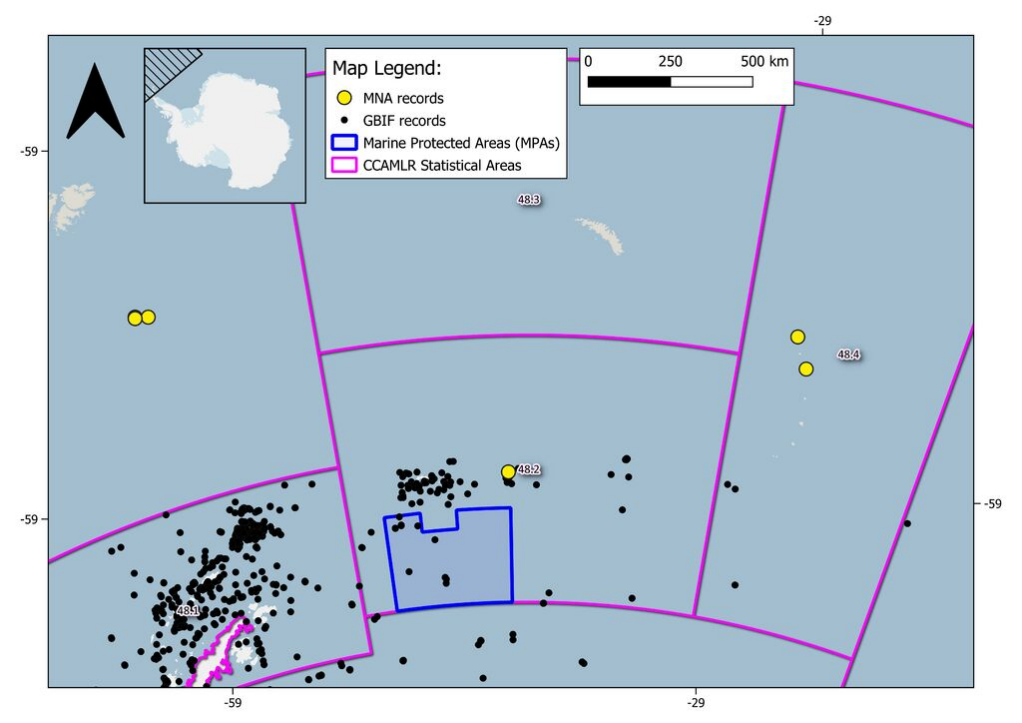
Focus of the South Orkney Islands and the South Georgia and South Sandwich Islands area records; Italian National Antarctic Museum (MNA, Section of Genoa) records are marked with yellow dots and Global Biodiversity Information System (GBIF) database (accessed on 10 October 2023) records are marked with black dots. CCAMLR (Convention on the Conservation of Antarctic Marine Living Resources) Statistical Areas, subareas and divisions are shown as purple lines, while Marine Protected Areas are represented as blue lines.

The geographical range of this study is the SO (Fig. 1). In this study, we specifically focused on the Ross Sea (RS), the South Orkney Islands (SOI) and the South Sandwich Islands (SSI) areas. The RS area is delimited by Cape Adare, located in the northern Victoria Land (West), to Cape Colbeck on the Edward VII Peninsula (East); as northern and southern delimitation, CCAMLR 88.1 and part of 88.2 Statistical subareas (CCAMLR 1980) are chosen (Figs 2, 3, 4). MNA samples retrieved in the SOI and the SSI areas are shown: CCAMLR 48.2 and 48.4 Statistical subareas are used in order to retrieve evidence of new records, using the GBIF data infrastructure respective datasets for the SOI and SSI (GBIF.Org 2023b, GBIF.Org 2023c) (Fig. 5). The RS CCAMLR 88.1 and 88.2 Statistical subareas are used for delimitation purposes, comparing records in the area using the SO Isopoda GBIF dataset (GBIF.Org 2023a), (Figs 2, 3). This dataset also includes 11 specimens belonging to the sub-antarctic Argentinean area of Burdwood Bank (Doti et al. 2020) (shown in Fig. 1).

Precise extension of the area of the samples is given in "Coordinates" section.

### Coordinates

−77.417 and −54.967 Latitude; −176.605 and 176.258 Longitude.

## Taxonomic coverage

### Description

**Figure 6.**
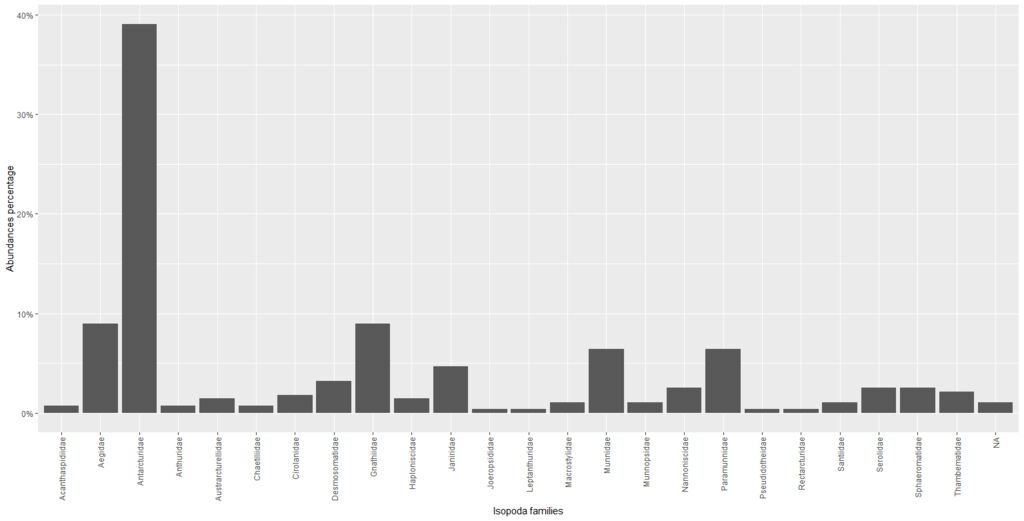
Percentages of the totality of the isopods specimens studied: NA represents unidentified isopods.

This dataset refers to a total of 279 sampling events from which 419 individuals were obtained, counted and identified at the lowest possible taxonomic level. Taxonomic identification led to the recognition of 81 species morphotypes, out of which 41 were classified at the species level, whereas the remaining 26 only at the genus level. Overall, the species found were distributed in 24 families and 51 genera. At the family level, the most abundant were Antarcturidae (37.71%), Munnidae (11.93%), Aegidae (7.88%), Santiidae (7.40%) and Gnathiidae (7.40%); Paramunnidae (4.53%), Janiridae (3.34%), Serolidae (2.63%), Desmosomatidae (2.63%), Thambematidae (1.67%), Nannoniscidae (1.67%), Sphaeromatidae (1.67%), Cirolanidae (1.43%), Chaetiliidae (1.43%), Macrostylidae (1.19%) are less represented; Austrarcturellidae (0.95%), Haploniscidae (0.95%), Munnopsidae (0.72%), Anthuridae (0.48%), Acanthaspidiidae (0.48%), Rectarcturidae (0.24%), Pseudidotheidae (0.24%), Leptanthuridae (0.24%) and Joeropsididae (0.24%) are the least represented families; unidentified isopods, named as "Isopoda" in this dataset, are the remaining 0.95% (Fig. 6).

In this checklist, 15 new records for RS area (marked with an asterisk) are presented: *Antarcturushodgsoni* Richardson, 1913, *Antarcturusstrasseni* Brandt, 1990, *Astrurusornatus* Vanhöffen, 1914, *Austroniscuschelus* Kaiser & Brandt, 2007, *Ceratoserolismeridionalis* (Vanhöffen, 1914), *Coperonusfrigida* (Vanhöffen, 1914), *Haploniscusantarcticus* Vanhöffen, 1914, *Holodentatacaeca* Doti, Choudhury & Brandt, 2009, *Joeropsiscurvicornis* (Nicolet, 1849), *Macrostylisroaldi* Riehl & Kaiser, 2012, *Mastigoniscuspolygomphios* Brökeland & Brandt, 2006, *Munnogoniumlongicaudatum* Just & Wilson, 2021, *Natatolanaparanarica* Keable, 2006, *Santiahispida* (Vanhöffen, 1914) and *Thambemathunderstruckae* Zemko & Kaiser, 2012. Out of these new records, five fall within the Ross Sea Marine Protected Area (Fig. 2) boundaries: *A.strasseni* Brandt, 1990, *C.frigida* (Vanhöffen, 1914), *M.roaldi* Riehl & Kaiser, 2012, *M.longicaudatum* Just & Wilson, 2021 and *S.hispida* (Vanhöffen, 1914).

For the SOI (CCAMLR statistical subarea 48.2) and SGSSI (CCAMLR statistical subarea 48.4) areas, any occurrence in this dataset is considered a new record.

### Taxa included

**Table:** 

Rank	Scientific Name	
family	Antarcturidae	
family	Anthuridae	
family	Desmosomatidae	
family	Sphaeromatidae	
genus	Acutiserolis Brandt, 1988	
genus	Aega Leach, 1816	
genus	Antarcturus zur Strassen, 1902	
genus	Austrofilius Hodgson, 1910	
genus	Austronanus Hodgson, 1910	
genus	Austroniscus Vanhöffen, 1914	
genus	Caecognathia Dollfus, 1901	
genus	Chaetarcturus Brandt, 1990	
genus	Desmosoma G. O. Sars, 1864	
genus	Exiliniscus Siebenaller & Hessler, 1981	
genus	Haploniscus Richardson, 1908	
genus	Ianthopsis Beddard, 1886	
genus	Mirabilicoxa Hessler, 1970	
genus	Moruloidea Baker, 1908	
genus	Munna Krøyer, 1839	
genus	Nannoniscus G. O. Sars, 1870	
genus	Palanana Just & Wilson, 2004	
genus	Pseudosphaeroma Chilton, 1909	
genus	Rectarcturus Schultz, 1981	
genus	Regabellator Siebenaller & Hessler, 1981	
genus	Thambema Stebbing, 1912	
genus	Torwolia Hessler, 1970	
kingdom	Coulmanniafrigida Hodgson, 1910	
order	Isopoda	
species	Acantharcturusbrevipleon Kussakin & Vasina, 1997	
species	Accalathuragigantissima Kussakin, 1967	
species	Acutiserolispoorei Brandt, 2009	
species	Aegiochusantarctica (Hodgson, 1910)	
species	Aegiochusglacialis (Tattersall, 1921)	
species	Antarcturusfurcatus (Studer, 1882)	
species	Antarcturushodgsoni Richardson, 1913*	
species	Antarcturuspolaris (Hodgson, 1902)	
species	Antarcturusspinacoronatus Schultz, 1978	
species	Antarcturusstrasseni Brandt, 1990*	
species	Astrurusornatus Vanhöffen, 1914*	
species	Austrofiliusfurcatus Hodgson, 1910	
species	Austroniscuschelus Kaiser & Brandt, 2007*	
species	Austrosignumglaciale Hodgson, 1910	
species	Caecognathiaantarctica (Studer, 1884)	
species	Caecognathiacalva (Vanhöffen, 1914)	
species	Caecognathiahodgsoni (Vanhöffen, 1914)	
species	Caecognathiapolaris (Hodgson, 1902)	
species	Ceratoserolismeridionalis (Vanhöffen, 1914)*	
species	Ceratoserolistrilobitoides (Eights, 1833)	
species	Chaetarcturusadareanus (Hodgson, 1902)	
species	Chaetarcturusbovinus (Brandt & Wägele, 1988)	
species	Chaetarcturuscervicornis Noli, Brandt, Di Franco & Schiaparelli, 2022	
species	Chaetarcturusfranklini (Hodgson, 1902)	
species	Cirolanamclaughlinae Bruce & Brandt, 2006	
species	Coperonusfrigida (Vanhöffen, 1914)*	
species	Dolichiscusgeorgei Kussakin & Vasina, 1980	
species	Dolichiscusmeridionalis (Hodgson, 1910)	
species	Euneognathiagigas (Beddard, 1886)	
species	Fissarcturuswalteri Brandt, 2013	
species	Glyptonotusacutus Richardson, 1906	
species	Haploniscusantarcticus Vanhöffen, 1914*	
species	Holodentatacaeca Doti, Choudhury & Brandt, 2009*	
species	Ianthopsisnasicornis Vanhöffen, 1914	
species	Ilyarachnaantarctica Vanhöffen, 1914	
species	Joeropsiscurvicornis (Nicolet, 1849)*	
species	Litarcturuslillei (Tattersall, 1921)	
species	Macrostylisroaldi Riehl & Kaiser, 2012*	
species	Mastigoniscuspolygomphios Brökeland & Brandt, 2006*	
species	Munnaantarctica (Pfeffer, 1887)	
species	Munnaglobicauda Vanhöffen, 1914	
species	Munnogoniumlongicaudatum Just & Wilson, 2021*	
species	Natatolanaintermedia (Vanhöffen, 1914)	
species	Natatolana meridionalis (Hodgson, 1910)	
species	Natatolanaparanarica Keable, 2006*	
species	Neojaeraantarctica (Pfeffer, 1887)	
species	Notopaisspicatus Hodgson, 1910	
species	Pagonanarostrata (Hodgson, 1910)	
species	Pseudidotheaarmata Noli, Di Franco, Schiaparelli & Brandt, 2022	
species	Santiacharcoti (Richardson, 1906)	
species	Santiahispida (Vanhöffen, 1914)*	
species	Thambemathunderstruckae Zemko & Kaiser, 2012*	

## Temporal coverage

**Data range:** 1995-1-23 – 2018-12-05.

## Collection data

### Collection name

MNA – Biological Collections

### Collection identifier

https://scientific-collections.gbif.org/collection/a57a1dc1-706c-42db-bbad-1e68d9685439

### Parent collection identifier

Italian National Antarctic Museum (MNA, Section of Genoa)

### Specimen preservation method

Absolute ethanol (96%); dried samples; frozen samples (−20°C).

## Usage licence

### Usage licence

Other

### IP rights notes

The dataset was published under the licence CC-BY 4.0.

## Data resources

### Data package title

Italian National Antarctic Museum (MNAIT) Antarctic Isopoda

### Resource link

https://doi.org/10.15468/w62z98

### Alternative identifiers

https://ipt.biodiversity.aq/resource?r=mna_isopoda

### Number of data sets

1

### Data set 1.

#### Data set name

Italian National Antarctic Museum (MNAIT) Antarctic Isopoda

#### Data format

Darwin Core

#### Description

This dataset is based on the distributional records obtained in the framework of 15 Antarctic Expeditions: X PNRA expedition (1994/95), XI (1995/96), XIII (1997/98), XIV (1998/99), XVII (2001/02), XIX (2003/04), XXV (2009/10), XXVII (2011/12), XXVIII (2012/13), XXIX (2013/14), XXXII (2016/17), NSF (National Science Foundation) "Icefish04", and BAS (British Antarctic Survey) expeditions JR15005 "SO-AntEco" and JR18003 "ICEBERGS2". All specimens belong to the National Antarctic Museum (MNA, Section of Genoa). This dataset will be useful to improve our knowledge of the isopods of the SO.

**Data set 1.** 

Column label	Column description
occurrenceID	A globally unique identifier for the Occurrence.
institutionCode	The name (acronym) in use by the institution having custody of the objects or information referred to in the record.
institutionID	An identifier for the institution having custody of the objects or information referred to in the record.
collectionCode	The acronym identifying the collection or dataset from which the record was derived.
collectionID	An identifier for the dataset from which the record was derived.
basisOfRecord	The specific nature of the data record (preserved specimen).
type	The genre of the resource.
scientificName	The full scientific name, with authorship and date information, if known.
taxonRank	The taxonomic rank of the most specific name in the scientificName.
kingdom	The full scientific name of the kingdom in which the taxon is classified.
phylum	The full scientific name of the phylum in which the taxon is classified.
class	The full scientific name of the class in which the taxon is classified.
order	The full scientific name of the order in which the taxon is classified.
family	The full scientific name of the family in which the taxon is classified.
genus	The full scientific name of the genus in which the taxon is classified.
specificEpithet	The name of the species epithet of the scientificName.
scientificNameAuthorship	The authorship information for the scientificName formatted according to the conventions of the applicable nomenclaturalCode.
identificationQualifier	A controlled value to express the determiner's doubts about the Identification.
scientificNameID	An identifier for the nomenclatural (not taxonomic) details of a scientific name.
individualCount	The number of individuals present at the time of the Occurrence.
typeStatus	A list (concatenated and separated) of nomenclatural types (type status, typified scientific name, publication) applied to the subject.
sex	The sex of the biological individual(s) represented in the Occurrence.
lifeStage	The age class or life stage of the Organism(s) at the time the Occurrence was recorded. Life stage "praniza" refers to larval stage of Gnathiidae Leach, 1814; life stage "manca" refers to postmarsupial stages (named "manca 1, 2, 3" according to the first, second and third moulting) of Isopoda Latreille, 1816.
occurrenceRemarks	Antarctic Expedition in which belongs the Occurrence.
fieldNumber	An identifier given to the event in the field and specific temporary identifier of the sample in the given event.
eventID	A global unique identifier for the set of information associated with an Event.
eventDate	The date-time or interval during which an Event occurred.
year	The four-digit year in which the Event occurred, according to the Common Era Calendar.
month	The integer month in which the Event occurred.
day	The integer day of the month on which the Event occurred.
decimalLatitude	The geographic latitude (in decimal degrees, using the spatial reference system given in geodeticDatum) of the geographic centre of a Location.
decimalLongitude	The geographic longitude (in decimal degrees, using the spatial reference system given in geodeticDatum) of the geographic centre of a Location. Positive values are east of the Greenwich Meridian, negative values are west of it.
geodeticDatum	The spatial reference system (SRS), upon which the geographic coordinates given in decimalLatitude and decimalLongitude are based.
minimumDepthInMetres	The lesser depth of a range of depth below the local surface, in metres.
maximumDepthInMetres	The greater depth of a range of depth below the local surface, in metres.
recordedBy	The person -or people- responsible for recording the original Occurrence.
recordedByID	A list (concatenated and separated) of the globally unique identifier for the person -or people- responsible for recording the original Occurrence.
identifiedBy	The name of the person who assigned the Taxon to the subject.
identifiedByID	The globally unique identifier of the person responsible for assigning the Taxon to the subject.
samplingProtocol	The methods or protocols used during an Event.
occurrenceStatus	A statement about the presence of a Taxon at a Location.
continent	The name of the continent in which the Location occurs.
countryCode	The standard code for the country in which the Location occurs.
preparations	A preparation or preservation method for a specimen.
catalogNumber	An unique identifier for the record within the dataset.
identificationRemarks	DOI referring the paper describing holotypes in the dataset.
coordinateUncertaintyInMetres	The horizontal distance (in metres) from the given dwc:decimalLatitude and dwc:decimalLongitude describing the smallest circle containing the whole of the dcterms:Location.
sampleSizeValue	A numeric value for a measurement of the size (time duration, length, area or volume) of a sample in a sampling.
sampleSizeUnit	The unit of measurement of the size of a sample in a sampling.

## References

[B11768793] Ahyong S., Boyko C. B., Bailly N., Bernot J., Bieler R., Brandão S. N., Daly M., De Grave S., Gofas S., Hernandez F., Hughes L., Neubauer T. A., Paulay G., Boydens B., Decock W., Dekeyzer S., Goharimanesh M., Vandepitte L., Vanhoorne B., Adlard R., Agatha S., Ahn K. J., Alvarez B., Amler M. R.W., Amorim V., Anderberg A., Andrés-Sánchez S., Ang Y., Antić D., Antonietto L. S.., Arango C., Artois T., Atkinson S., Auffenberg K., Baldwin B. G., Bank R., Barber A., Bartsch I., Bellan-Santini D., Bergh N., Berta A., Bezerra T. N., Blanco S., Blasco-Costa I., Blazewicz M., Błędzki L. A., Bock P., Bonifacino M., Böttger-Schnack R., Bouchet P., Boury-Esnault N., Bouzan R., Boxshall G., Bray R., Brito Seixas A. L., Bruce N. L., Bruneau A., Budaeva N., Bueno-Villegas J., Calvo Casas J., Cárdenas P., Carstens E., Cartwright P., Cedhagen T., Chan B. K., Chan T. Y., Choong H., Christenhusz M., Churchill M., Collins A. G., Collins G. E., Collins K., Consorti L., Copilaș-Ciocianu D., Corbari L., Cordeiro R., Costa V. M.d.M., Costa Corgosinho P. H., Coste M., Costello M. J., Crandall K. A., Cremonte F., Cribb T., Cutmore S., Dahdouh-Guebas F., Daneliya M., Dauvin J. C., Davie P., De Broyer C., de Lima Ferreira P., de Mazancourt V., de Moura Oliveira L., de Sá H. A.B.., de Voogd N. J., Decker P., Defaye D., Dekker H., d'Hondt J. L., Di Capua I., Dippenaar S., Dohrmann M., Dolan J., Domning D., Downey R., Dreyer N., Eisendle U., Eitel M., Eleaume M., Enghoff H., Epler J., Esquete Garrote P., Evenhuis N. L., Ewers-Saucedo C., Faber M., Figueroa D., Fišer C., Fordyce E., Foster W., Fransen C., Freire S., Fujimoto S., Furuya H., Galbany-Casals M., Gale A., Galea H., Gao T., Garic R., Garnett S., Gaviria-Melo S., Gerken S., Gibson D., Gibson R., Gil J., Gittenberger A., Glasby C., Glenner H., Glover A., Gómez-Noguera S. E., Gondim A. I., Gonzalez B. C., González-Solís D., Goodwin C., Gostel M., Grabowski M., Gravili C., Grossi M., Guerra-García J. M.., Guerrero J. M., Guidetti R., Guiry M. D., Gutierrez D., Hadfield K. A., Hajdu E., Halanych K., Hallermann J., Hayward B. W., Hegna T. A., Heiden G., Hendrycks E., Hennen D., Herbert D., Herrera Bachiller A., Hodda M., Høeg J., Hoeksema B., Holovachov O., Hooge M. D., Hooper J. N., Horton T., Houart R., Huys R., Hyžný M., Iniesta L. F.M., Iseto T., Iwataki M., Janssen R., Jaume D., Jazdzewski K., Jersabek C. D., Jiménez-Mejías P., Jóźwiak P., Kabat A., Kakui K., Kantor Y., Karanovic I., Karapunar B., Karthick B., Kathirithamby J., Katinas L., Kilian N., Kim Y. H., King R., Kirk P. M., Klautau M., Kociolek J. P., Köhler F., Konowalik K., Kotov A., Kovács Z., Kremenetskaia A., Kristensen R. M., Kroh A., Kulikovskiy M., Kullander S., Kupriyanova E., Lamaro A., Lambert G., Laridon I., Lazarus D., Le Coze F., Le Roux M., LeCroy S., Leduc D., Lefkowitz E. J., Lemaitre R., Lichter-Marck I. H., Lim S. C., Lindsay D., Liu Y., Loeuille B., Lörz A. N., Ludwig T., Lundholm N., Macpherson E., Mah C., Mamos T., Manconi R., Mapstone G., Marek P. E., Markello K., Marshall B., Marshall D. J., Martin P., Martinez Arbizu P., McFadden C., McInnes S. J., McKenzie R., Means J., Mees J., Mejía-Madrid H. H., Meland K., Merrin K. L., Miller J., Mills C., Moestrup Ø., Mokievsky V., Molodtsova T., Monniot F., Mooi R., Morandini A. C., Moreira da Rocha R., Morrow C., Mortelmans J., Müller A., Muñoz Gallego A. R., Musco L., Nascimento A. L.D.S., Nascimento J. B., Nesom G., Neto Silva M. d.S., Neubert E., Neuhaus B., Ng P., Nguyen A. D., Nielsen C., Nielsen S., Nishikawa T., Norenburg J., O'Hara T., Opresko D., Osawa M., Osigus H. J., Ota Y., Páll-Gergely B., Panero J. L., Patterson D., Pedram M., Pelser P., Peña Santiago R., Pereira J. d.S.., Pereira S. G.G., Perez-Losada M., Petrescu I., Pfingstl T., Piasecki W., Pica D., Picton B., Pignatti J., Pilger J. F., Pinheiro U., Pisera A. B., Poatskievick Pierezan B., Polhemus D., Poore G. C., Potapova M., Praxedes R. A., Půža V., Read G., Reich M., Reimer J. D., Reip H., Resende Bueno V., Reuscher M., Reynolds J. W., Richling I., Rimet F., Ríos P., Rius M., Rodríguez E., Rogers D. C., Roque N., Rosenberg G., Rützler K., Saavedra M., Sabbe K., Sabroux R., Saiz-Salinas J., Sala S., Samimi-Namin K., Santagata S., Santos S., Santos S. G., Sanz Arnal M., Sar E., Saucède T., Schärer L., Schierwater B., Schilling E., Schmidt-Lebuhn A., Schneider S., Schönberg C., Schrével J., Schuchert P., Schweitzer C., Semple J. C., Senna A. R., Sennikov A., Serejo C., Shaik S., Shamsi S., Sharma J., Shear W. A., Shenkar N., Short M., Sicinski J., Sidorov D., Sierwald P., Silva D. K.F.d., Silva E. S.S., Silva M. L.C.N., Simmons E., Sinniger F., Sinou C., Sivell D., Smit H., Smit N., Smol N., Sørensen M. V., Souza-Filho J. F.., Spelda J., Sterrer W., Steyn H. M., Stoev P., Stöhr S., Suárez-Morales E., Susanna A., Suttle C., Swalla B. J., Taiti S., Tanaka M., Tandberg A. H., Tang D., Tasker M., Taylor J., Taylor J., Taylor K., Tchesunov A., Temereva E., ten Hove H., ter Poorten J. J., Thirouin K., Thomas J. D., Thuesen E. V., Thurston M., Thuy B., Timi J. T., Todaro A., Todd J., Turon X., Uetz P., Urbatsch L., Uribe-Palomino J., Urtubey E., Utevsky S., Vacelet J., Vader W., Väinölä R., Valls Domedel G., Van de Vijver B., van der Meij S. E., van Haaren T., van Soest R. W., Vanreusel A., Venekey V., Verhoeff T., Vinarski M., Vonk R., Vos C., Vouilloud A. A., Walker-Smith G., Walter T. C., Watling L., Wayland M., Wesener T., Wetzel C. E., Whipps C., White K., Wieneke U., Williams D. M., Williams G., Wilson R., Witkowski J., Wyatt N., Xavier J., Xu K., Zanol J., Zeidler W., Zhao Z., Zullini A. (2024). World Register of Marine Species (WoRMS). https://www.marinespecies.org.

[B10563835] Barnes David K. A. (2008). A benthic richness hotspot in the Southern Ocean: slope and shelf cryptic benthos of Shag Rocks. Antarctic Science.

[B11103862] Barnes David K. A., Kaiser Stefanie, Griffiths Huw J., Linse Katrin (2009). Marine, intertidal, freshwater and terrestrial biodiversity of an isolated polar archipelago. Journal of Biogeography.

[B11103850] Berkman Paul, Cattaneo-Vietti Riccardo, Chiantore Mariachiara, Howard-Williams Clive, Cummings V., Kvitek Rikk (2005). Marine research in the Latitudinal Gradient Project along Victoria Land, Antarctica. Scientia Marina.

[B9524294] Bonello Guido, Grillo Marco, Cecchetto Matteo, Giallain Marina, Granata Antonia, Guglielmo Letterio, Pane Luigi, Schiaparelli Stefano (2020). Distributional records of Ross Sea (Antarctica) planktic Copepoda from bibliographic data and samples curated at the Italian National Antarctic Museum (MNA): checklist of species collected in the Ross Sea sector from 1987 to 1995. ZooKeys.

[B9382215] Brandt Angelika (1999). On the origin and evolution of Antarctic Peracarida (Crustacea, Malacostraca). Scientia Marina.

[B11105939] Brandt Angelika, Linse Katrin, Mühlenhardt-Siegel Ute (1999). Biogeography of Crustacea and Mollusca of the Subantarctic and Antarctic regions. Scientia Marina.

[B9382247] Brandt Angelika, Brökeland Wiebke, Brix Saskia, Malyutina Marina (2004). Diversity of Southern Ocean deep-sea Isopoda (Crustacea, Malacostraca) — a comparison with shelf data. Deep Sea Research Part II: Topical Studies in Oceanography.

[B9382121] Broyer Claude De, Jazdzewski Krzysztof, Dauby Patrick (2003). Biodiversity patterns in the Southern Ocean: Lessons from Crustacea. Antarctic Biology in a Global Context.

[B10560690] CCAMLR (1980). V.D.4 Convention on the Conservation of Antarctic Marine Living Resources (CCAMLR) (20 May 1980). International Law & World Order.

[B9524342] Cecchetto Matteo, Alvaro Maria Chiara, Ghiglione Claudio, Guzzi Alice, Mazzoli Claudio, Piazza Paola, Schiaparelli Stefano (2017). Distributional records of Antarctic and sub-Antarctic Ophiuroidea from samples curated at the Italian National Antarctic Museum (MNA): check-list update of the group in the Terra Nova Bay area (Ross Sea) and launch of the MNA 3D model ‘virtual gallery’. ZooKeys.

[B9524380] Cecchetto Matteo, Lombardi Chiara, Canese Simonepietro, Cocito Silvia, Kuklinski Piotr, Mazzoli Claudio, Schiaparelli Stefano (2019). The Bryozoa collection of the Italian National Antarctic Museum, with an updated checklist from Terra Nova Bay, Ross Sea. ZooKeys.

[B9722518] Chamberlain Scott, Oldoni Damiano, Waller John rgbif: interface to the global biodiversity information facility API. https://CRAN.R-project.org/package=rgbif..

[B9477409] Choudhury Madhumita, Brandt Angelika (2007). Composition and distribution of benthic isopod (Crustacea, Malacostraca) families off the Victoria-Land Coast (Ross Sea, Antarctica). Polar Biology.

[B9477418] Choudhury Madhumita, Brandt Angelika (2009). Benthic isopods (Crustacea, Malacostraca) from the Ross Sea, Antarctica: species checklist and their zoogeography in the Southern Ocean. Polar Biology.

[B9382234] Clarke Andrew, Johnston Nadine M., Gibson R. N., Atkinson R. J. A. (2003). Oceanography and Marine Biology, An Annual Review, Volume 41.

[B11243874] USA Delegations of New Zealand and the (2016). A proposal for the establishment of a Ross Sea Region marine protected area. https://meetings.ccamlr.org/en/ccamlr-xxxv/25-rev-1.

[B11769234] Di Franco Davide, Linse Katrin, Griffiths Huw J., Haas Christian, Saeedi Hanieh, Brandt Angelika (2020). Abundance and Distributional Patterns of Benthic Peracarid Crustaceans From the Atlantic Sector of the Southern Ocean and Weddell Sea. Frontiers in Marine Science.

[B11765459] Doti Brenda L., Chiesa Ignacio L., Roccatagliata Daniel (2020). Biodiversity of Isopoda and Cumacea (Peracarida, Crustacea) from the Marine Protected Area Namuncurá-Burdwood Bank, South-West Atlantic. Polar Biology.

[B9524392] Garlasché Giuseppe, Karimullah Karimullah, Iakovenko Nataliia, Velasco-Castrillón Alejandro, Janko Karel, Guidetti Roberto, Rebecchi Lorena, Cecchetto Matteo, Schiaparelli Stefano, Jersabek Christian D., De Smet Willem H., Fontaneto Diego (2019). A data set on the distribution of Rotifera in Antarctica. Biogeographia – The Journal of Integrative Biogeography.

[B10561380] GBIF.Org (2023). Occurrence Download. https://www.gbif.org/occurrence/download/0012812-230224095556074.

[B10560701] GBIF.Org (2023). Occurrence Download. https://www.gbif.org/occurrence/download/0000298-231002084531237.

[B10561398] GBIF.Org (2023). Occurrence Download. https://www.gbif.org/occurrence/download/0000305-231002084531237.

[B9524354] Ghiglione Claudio, Alvaro Maria Chiara, Griffiths Huw, Linse Katrin, Schiaparelli Stefano (2013). Ross Sea Mollusca from the Latitudinal Gradient Program: R/V Italica 2004 Rauschert dredge samples. ZooKeys.

[B9524364] Ghiglione Claudio, Alvaro Maria Chiara, Cecchetto Matteo, Canese Simonepietro, Downey Rachel, Guzzi Alice, Mazzoli Claudio, Piazza Paola, Tore Rapp Hans, Sarà Antonio, Schiaparelli Stefano (2018). Porifera collection of the Italian National Antarctic Museum (MNA), with an updated checklist from Terra Nova Bay (Ross Sea). ZooKeys.

[B11238137] Grillo Marco, Bonello Guido, Cecchetto Matteo, Guzzi Alice, Noli Nicholas, Cometti Valentina, Schiaparelli Stefano (2024). Planktonic, benthic and sympagic copepods collected from the desalination unit of Mario Zucchelli Research Station in Terra Nova Bay (Ross Sea, Antarctica). Biodiversity Data Journal.

[B9476596] Guzzi Alice, Alvaro Maria Chiara, Danis Bruno, Moreau Camille, Schiaparelli Stefano (2022). Not all that glitters is gold: Barcoding effort reveals taxonomic incongruences in iconic Ross Sea Sea Stars. MDPI Diversity.

[B11150779] UNESCO Intergovernmental Oceanographic Commission of (2023). Ocean Biodiversity Information System. https://obis.org/.

[B10563959] Kaiser Stefanie, Barnes David K. A., Linse Katrin, Brandt Angelika (2008). Epibenthic macrofauna associated with the shelf and slope of a young and isolated Southern Ocean island. Antarctic Science.

[B10563844] Kaiser Stefanie, Barnes David K. A., Sands Chester J., Brandt Angelika (2009). Biodiversity of an unknown Antarctic Sea: assessing isopod richness and abundance in the first benthic survey of the Amundsen continental shelf. Marine Biodiversity.

[B11109809] Kaiser Stefanie, Broyer Claude De, Koubbi Philippe, Griffiths Huw, Raymond Ben, d’Acoz Cédric d’Udekem, Putte Anton Van de, Danis Bruno, David Bruno, Grant Susie, Gutt Julian, Held Christoph, Hosie Graham, Huettmann Falk, Post Alix, Ropert-Coudert Yan (2014). Biogeographic Atlas of the Southern Ocean.

[B9382049] Kussakin O. G. (1967). Fauna of Isopoda and Tanaidacea in the coastal zones of the Antarctic and Subantarctic waters, in•. Biological Report of the Soviet Antarctic Expeditions 1955-1958, 3. Issled. Fauny Morei.

[B9171525] Matsuoka Kenichi, Skoglund Anders, Roth George, de Pomereu Jean, Griffiths Huw, Headland Robert, Herried Brad, Katsumata Katsuro, Le Brocq Anne, Licht Kathy, Morgan Fraser, Neff Peter D., Ritz Catherine, Scheinert Mirko, Tamura Takeshi, Van de Putte Anton, van den Broeke Michiel, von Deschwanden Angela, Deschamps-Berger César, Van Liefferinge Brice, Tronstad Stein, Melvær Yngve (2021). Quantarctica, an integrated mapping environment for Antarctica, the Southern Ocean, and sub-Antarctic islands. Environmental Modelling & Software.

[B10563875] Noli Nicholas, Di Franco Davide, Schiaparelli Stefano, Brandt Angelika (2022). *Pseudidotheaarmata* sp. n., a new isopod of the genus *Pseudidothea* (Crustacea, Malacostraca, Isopoda) from the Atlantic sector of the Southern Ocean. Biodiversity Data Journal.

[B9524317] Piazza Paola, Blazewicz-Paszkowycz Magdalena, Ghiglione Claudio, Alvaro Maria Chiara, Schnabel Kareen, Schiaparelli Stefano (2014). Distributional records of Ross Sea (Antarctica) Tanaidacea from museum samples stored in the collections of the Italian National Antarctic Museum (MNA) and the New Zealand National Institute of Water and Atmospheric Research (NIWA). ZooKeys.

[B9477399] Rehm Peter, Thatje Sven, Arntz Wolf E., Brandt Angelika, Heilmayer Olaf (2006). Distribution and composition of macrozoobenthic communities along a Victoria-Land Transect (Ross Sea, Antarctica). Polar Biology.

[B9524328] Selbmann Laura, Onofri Silvano, Zucconi Laura, Isola Daniela, Rottigni Marino, Ghiglione Claudio, Piazza Paola, Alvaro Maria Chiara, Schiaparelli Stefano (2015). Distributional records of Antarctic fungi based on strains preserved in the Culture Collection of Fungi from Extreme Environments (CCFEE) Mycological Section associated with the Italian National Antarctic Museum (MNA). MycoKeys.

